# Cytosine Methyltransferases as Tumor Markers

**DOI:** 10.2174/138920210793360916

**Published:** 2010-12

**Authors:** Athanasia Pavlopoulou, Sophia Kossida

**Affiliations:** 1 Biomedical Research Foundation of the Academy of Athens, Department of Biotechnology, Bioinformatics & Medical Informatics Team, Soranou Efesiou 4, 11527 Athens, Greece; 2 Department of Pharmacy, School of Health Sciences, University of Patras, GR-26500 Rion-Patras, Greece

**Keywords:** Biomarkers, cancer, chromatin modification, CpG methylation, DNMTs, epigenetic therapy, oncogenes, tumor suppressor genes.

## Abstract

Changes in DNA methylation patterns is a prominent characteristic of human tumors. Tumor cells display reduced levels of genomic DNA methylation and site-specific CpG island hypermethylation. Methylation of CpG dinucleotides is catalyzed by the enzyme family of DNA methyltransferases (DNMTs). In this review, the role of DNA methylation and DNMTs as key determinants of carcinogenesis is further elucidated. The chromatin modifying proteins that are known to interact with DNMTs are also described. Finally, the role of DNMTs as potential therapeutic targets is addressed.

## 1. INTRODUCTION

Cancer has long been viewed as a set of diseases caused by progressive genetic and epigenetic alterations. However, a large and expanding body of evidence suggests that the initiation and progression of several cancer types is controlled by epigenetic modifications-heritable changes in gene expression patterns that are mediated by mechanisms other than changes in the primary DNA sequence. Since epigenetic modifications are reversible, as opposed to genetic lesions, much research has been invested in their characterization . Aberrant DNA methylation is the most prominent form of epigenetic modification in cancer [1-3]. DNA methylation is caused by the covalent addition of a methyl group at the 5’ position of the pyrimidine ring of cytosines within the context of CpG dinucleotides [4]. In human malignant cells, aberrant CpG methylation patterns are accompanied by genome-wide hypomethylation and gene-specific hypermethylation [3, 5]. The tumor CpG methylation profiles are therefore useful biomarkers for cancer detection and molecular classification of the various cancer types. The aberrant CpG methylation patterns in human cancer cells are inscribed by the *de novo* DNA cytosine-5 methyltransferases (DNMTs), DNMT3a and DNMT3b, and transmitted in the subsequent cell generations by the maintenance DNMT1 [4, 6]. DNMTs through interactions with chromatin modifying enzymes result in a compact chromatin structure which inhibits gene tanscription.

In the current paper, a comprehensive review of CpG methylation, DNA methyltransferases and cancer is presented. Taking into consideration previous reports on this subject, an update is provided in order to further enhance our understanding regarding the major epigenetic mechanism that contributes to carcinogenesis. The DNA methylation mechanism and the DNA methylation machinery are described in detail along with the associations between DNMTs and chromatin modifying protein complexes. 

## 2. ABERRANT CpG METHYLATION PATTERNS IN CANCER

Aberrant methylation of CpG dinucleotides is an early event in carcinogenesis which increases in magnitude during tumor progression. DNA hypermethylation would mediate most of the important pathway anomalies in cancer including genetic instability, disruption of normal cell-cell interaction, inactivation of signal transduction cascades, loss of apoptotic signals etc. [7, 8].

### 2.1. CpG Island Methylation and Epigenetic Gene Silencing

A characteristic of the mammalian DNA methylation patterns is the presence of CpG islands, regions with a high C and G content and a frequent occurrence of CpG dinucleotide clusters. CpG islands are usually positioned near the transcription start sites of expressed genes [9, 10]. Several lines of evidence suggest an inverse correlation between methylation of CpG dinucleotides and transcriptional gene regulation. How does cytosine-5 DNA methylation lead to transcriptional silencing? 

**Fig. (1):**
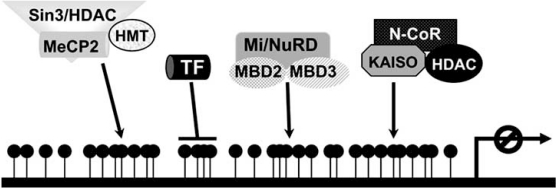
Mechanisms of transcriptional inactivation by cytosine-5 methylation. The binding of transcription factors (TFs) to their cognate sequences is prevented by DNA methylation. Protein complexes recruited by DNA methylation establish a condensed chromatin sctructure which renders the template inaccessible to the cellular transcription apparatus. DNA is represented by a thick black line. The methylated cytosine residues are shown as filled black lollipops.

According to the first mechanism, the 5-methyl cytosine protrudes into the major groove of the DNA double helix thereby preventing the binding of transcription factors or the basal transcriptional machinery to their recognition sites (Fig. **1**) [11].

The second mechanism that contributes to methylation-induced transcriptional silencing involves specific proteins that selectively bind to methylated CpG dinucleotides [12]. These proteins include a conserved family of proteins that share a common motif, the methyl-CpG-binding domain (MBD) [13]. Four members of this family, MBD1-3 [14] and the mammalian methyl CpG-binding protein 2 (MeCP2) [15] are implicated in DNA methylation-dependent transcriptional silencing [16-18]. The founding member of this family, MeCP2, can inhibit transcription through a minimal transcriptional repression domain (TRD) [19]. MeCP2 is also capable of binding to abundant sites both in naked DNA and within chromatin, as well as interacting with several regulatory proteins [15]. The methyl-CpG binding proteins possibly cause methylation-mediated transcriptional repression by excluding certain transcription factors from binding to their recognition sites (Fig. **1**). 

Dense methylation of the CpG islands of the promoter regions of cancer-associated genes has been reported to lead to a transcriptionally inactive, compact chromatin structure [20]. The chromatin is organized in a compressed form, representing an obstacle to RNA polymerase and to regulatory DNA-binding proteins to access the eukaryotic promoters. At sites where transcriptional activation occurs, this tightly coiled and condensed chromatin structure unfolds and the DNA is sensitive to cleavage by DNase I; this condensed chromatin structure is maintained at sites where transcription is repressed [21]. DNA is wrapped around an octamer of histone proteins, [H2A-H2B]2-[H32-H42], to form the nucleosome, which is the fundamental repeating unit of chromatin [22]. Therefore, histone modification is a powerful device to unfold chromatin. Most histone modifications occur in the lysine residues in their protruding N-terminal tail domains which are subject to modifications, such as acetylation and methylation [23].

Acetylation of histones H3 and H4 by histone acetyltransferases (HATs) weakens electrostatic DNA-histone interactions and promotes an open chromatin conformation. Acetylated histone tails increase the affinity of chromatin for transcription factors and bromodomain-containing proteins, generally leading to transcriptional activation [24]. Conversely, deacetylation of histones by HDACs (histone deacetylases) strongly correlates with chromatin condensation and transcriptional repression [25]. The other histone modification, histone methylation, is catalyzed by the enzyme family of histone methyltransferases (HMTs). Histone methylation can either result in transcriptional activation or repression, depending on the target lysine. For instance, methylation of histone lysines H3-K9, H3-K27 and H4-K20 generally correlates with transcriptional gene silencing, whereas H3-K4, H3-K36 and H3-K27 methylation is usually associated with transcriptional gene activation [26, 27]. 

The two epigenetic processes, DNA methylation and chromatin modification, are dynamically linked in order to silence genes in cancer. As support to this notion, several MBD proteins are shown to be associated with histone modifying complexes (Fig. **1**). For example, the mammalian MeCP2 was shown to be associated with large protein complexes containing the transcriptional co-repressor Sin3A which in turn binds the histone deacetylases HDAC1 and HDAC2 [28, 29], as well as a histone H3K9-specific HMT [30]. Besides, MeCP2 has been shown to be associated with Brahma, a catalytic domain of a SWI/SNF–like ATP-dependent chromatin remodeling complex that disrupts DNA-histone interactions. This association led to transcriptional inactivation [31]. Furthermore, the protein MBD2 belongs to the MeCP1 complex together with the HDAC1 and HDAC2, and two retinoblastoma-associated histone-binding proteins [32]. MBD3 was identified as a core component of the Mi2-NuRD complex, which exhibits both histone deacetylase and ATPase-dependent nucleosome remodeling activities. MBD2 interacts with MBD3 to recruit the Mi2-NuRD complex to methylated DNA [33, 34] (see Fig. **1**). 

Kaiso is another protein able to selectively bind to methyl CpG, although it lacks the MBD motif [35]. Kaiso is a component of the human HDAC-containing co-repressor complex, N-CoR, which resembles the Sin3 and NuRD complex [36, 37]. N-CoR is implicated in the transcriptional repression of a vast number of transcriptional factors [38]. The Kaiso/N-CoR complex was demonstrated to be able to cause DNA methylation-mediated transcriptional repression involving both hypoacetylation and H3-K9 methylation (Fig. **1**) [39]. Furthermore, Kaiso is a member of the BTB/POZ family of zinc finger transcription factors, which includes the oncogenic proteins BCL, ETO and PLZF [40]. It would be intriguing to further investigate if other members of this family have methyl-CpG binding activity. 

Additional proof that DNA methylation and chromatin modifications are functionally connected comes from the human *ATRX* [41] and the murine *lymphoid-specific* *helicase 2(Lsh2)* genes [42], both of which encode homologues of the chromatin remodeling protein SNF2. Mutations in these genes led to diverse changes in the global DNA methylation patterns [41, 42].

### 2.2. A Direct Role for CpG Island Methylation in Carcinogenesis

**Fig. (2):**
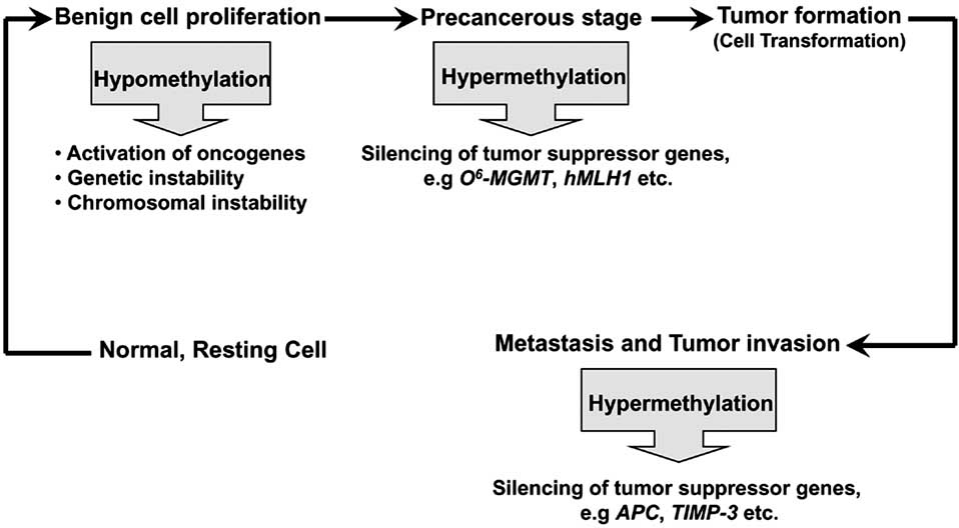
Model of epigenetic regulation in tumorigenesis.

The genome of tumor cells is characterized by profound alterations of DNA methylation compared to their normal counterparts. Whether CpG island methylation is a cause or a consequence of cancer is a controversial issue. The early occurrence of CpG methylation in several cancer types is suggestive of a critical role in the etiology of human cancer. Typically, tumor cells exhibit genome-wide CpG dinucleotide hypomethylation concomitant with CpG hypermethylation of tumor suppressor and pro-apoptotic genes (Fig. **2**) [43, 44].

#### 2.2.1. CpG Island Hypermethylation in Cancer is Widespread

Ample evidence suggests that CpG islands in the promoters of various tumor suppressor genes are frequently hypermethylated in cancer cells resulting in gene silencing [8, 44]. Abnormal gene silencing occurs more frequently during the early stages of tumorigenesis such as the precancerous stages of tumor development (Fig. **2**) [45]. Several studies have reported abnormal epigenetic silencing of several genes during the pre-invasive stages of lung, prostate and colon cancer. These early epigenetic changes could predispose cells to genetic abnormalities that promote tumorigenesis [1]. 

Disruption of the function of a tumor-suppressor gene, according to Knudson’s two-hit hypothesis, requires complete loss of function of both gene copies [46]. The first hit involves germline mutation (in familial cancer) or somatic mutation (in non-inherited cancers). On the contrary, the second hit generally involves epigenetic inactivation of the wild-type allele of a tumor suppressor gene by aberrant promoter hypermethylation which leads to complete disruption of the function of the gene [47]. Hypermethylation-mediated gene silencing can lead to selective loss of key gene functions in cancer [47, 48]. A brief list of the most important genes inactivated by promoter hypermethylation in cancer along with the cellular pathways involved is presented in Table **1**.

Hypermethylated genes were identified that are shared within each tumor type and others that exhibited distinct tumor-type-specificity [5]. For example, the gene encoding the cyclin-dependent kinase inhibitor *p16^INK4a^* which is required in the cyclinD-Rb pathway is disrupted in virtually all tumors [49]. Moreover, p53 is considered as the tumor-suppressor gene most commonly mutated in cancer [48]. On the other hand, certain tumor-suppressor genes are silenced in different tumor types. In particular, promoter hypermethylation-induced transcriptional inactivation of the *BRCA1*, *RASSF1A* and *GSTP1* genes occurs almost exclusively in familial breast [50], lung [51] and prostate cancer [52], respectively. In addition, hypermethylation of the *estrogen-receptor (ER)* gene in ageing colorectal mucosa results in predisposition to sporadic colorectal carcinogenesis [53].

Candidate tumor suppressor genes can be identified by virtue of promoter hypermethylation. According to Laird [54], dense hypermethylation of CpG islands provides one of the most promising markers for early cancer detection and risk assessment for the future development of disease. The efficiency of a biomarker assay is determined by its sensitivity (the minimal quantity of the substrate that can be detected) and specificity (the percentage of assays that correctly distinguish normal from cancer-containing samples) [55]. The restriction landmark genomic scanning (RLGS) assay is one of the earliest tools that have been utilized to search for genome-wide aberrant CpG island methylation patterns [56]. Sensitive detection of aberrant CpG methylation patterns in cancer relies on the principle of MSP (methylation-specific PCR) [57] or fluorescent-based variants, such as MethyLight [58]. Another suitable assay for high-throughput CpG methylation analysis in cancer is methylation-specific oligonucleotide (MSO) microarray method. Following bisulfite treatment and PCR amplification, the products are hybridized on microarrays. MSO microarray is able to detect methylation at specific nucleotide positions. Fluorescence detection is used to obtain quantitative differences [59].

Prediction of response to a therapeutic treatment is another potential application of CpG-island hypermethylation as biomarker [54]. For instance, transcriptional inactivation of the DNA repair genes *hMLH1* and *O^6^-MGMT* by hypermethylation resulted in microsatellite instability [60] and increased frequency of mutation rates [61], respectively owing to faulty DNA repair. O6-MGMT is responsible for repairing acetylation damage to the guanine base of DNA. Thus, tumors with defective O6-MGMT function due to hypermethylation would be more sensitive to the effects of chemotherapy that depends on alkylating agents because of the diminished capacity of cancer cells to repair DNA lesions which leads to cell death [61]. 

#### 2.2.2. DNA Hypomethylation of Transformed Cells 

According to Esteller [43], the degree of the undermethylated genomic DNA increases through all the stages of tumorigenesis, from the benign proliferations to the invasive cancers (Fig. **2**). DNA hypomethylation can contribute to tumorigenesis by invoking two mechanisms. The first one is the potentially harmful re-activation of previously silent proteo-oncogenes, such as *Raf*, *c-Myc*, *c-H-RAs*, *c-K-Ras*, *c-Fos* and *BCL-2* [62]. Hypomethylation of oncogenes could alleviate the transcriptional repression imposed by methylation in the promoter region of these genes and restore their expression. 

The second mechanism by which DNA deficiency is suggested to play a significant causal role in oncogenesis is by promoting genomic and chromosomal instability. The main purpose of DNA methylation has been proposed to be genome control by repressing the transcription of parasitic and mobile DNA elements [63]. Undermethylation of malignant cell DNA can reactivate intragenomic parasitic DNA elements, such as ALU and LINES [64, 65]. These and other normally silent transposable elements pose a threat to genomic integrity by inserting themselves into coding regions, disrupting the active host gene or modulating its regulated expression [66]. Transposons favour recombination between non-allelic repeats that can cause unbalanced translocations, deletions or isochromosome formation [64]. DNA methylation may suppress recombination either by occluding the recombination initiation site or by methylating the recombination-associated genes and inhibiting their transcription [67].

DNA deficiency leads to chromosome instability, as well, which is the most common form of genetic instability in human cancer [68]. The maintenance of proper DNA methylation in the heterochromatin in the vicinity of the centromere (pericentromeric region) - the site of attachment of the mitotic spindle - is a prerequisite for stability and for faithful DNA replication [47]. DNA hypomethylation has been associated with the rare recessive disease ICF (Immunodeficiency, Centromeric instability, Facial anomalies). The most diagnostic feature of this disease is profound pericentromeric rearrangements in mitogen-stimulated lymphocytes [69, 70]. Similarly, some of these pericentromeric regions have been frequently observed in many types of cancer. In particular, it was found that 15-40% of breast adenocarcinomas display hypomethylation of the classical satellite DNA (the major components of the constitutive heterochromatin) at the pericentromeric regions along with pericentromeric rearrangements [71]. Chromosome instability is more pronounced in stimulated thymic lymphocytes in mice, accompanied by extensive loss of DNA methylation; the clinical manifestation is combined immunodeficiency. Thymic lymphocytes may express factors that destabilize undermethylated DNA or fail to express factors that stabilize hypomethylated sequences [72, 73].

## 3. THE DNA METHYLATION MACHINERY

Aberrant CpG-island methylation in cancer is regulated by DNMT1, DNMT3A and DNMT3B which catalyze the transfer of a methyl moiety from the methyl donor *S*-adenosyl-L-methionine to the carbon-5 of a cytosine base [4, 6]. DNMTs consist of the catalytic C- terminal domain and several regulatory domains in the N terminus [6]. DNMT1 has a preference for hemimethylated DNA and is considered responsible for maintaining the DNA methylation patterns [4, 6, 74]. DNMT3A and DNMT3B mediate *de novo* methylation during embryogenesis and development [4, 6, 75]. However, this distinction of functions is not clear, since DNMT3A and DNMT3B are involved in maintenance methylation [76] and DNMT1 can contribute to *de novo* methylation in specific cases [6, 77]. 

### 3.1. A Direct Role for DNMTs in Tumorigenesis

Numerous studies implicate DNMTs in the DNA abnormalities in cancer. Constitutive over-expression of exogenous DNMT1 in tissue culture cells was reported to induce a cell passage-dependent, gradual, hypermethylation of selected CpG island which was accompanied by tumorigenic transformation [77, 78]. Moreover, overexpression of *DNMT1* contributes to cell transformation by the *fos* oncogene [79]. Increased levels of DNMTs and activities occur in various types of cancer. It was shown that a reduction in the *Dnmt1* activity due to heterozygosity or treatment of mice with the Dnmt1 inhibitor 5-aza-2’-deoxycytidine, reduced the number of intestinal polypops in mice heterozygous for *Apc^Min^*, a mutation that predisposed to colonal neoplasia [80, 81]. DNMT1 was also reported to be overexpressed in human colon cancer (several hundredfold) in comparison to normal colon mucosa [82]. Moreover, elevated DNMT1 protein levels were observed in MCF-7 human breast cancer cells compared to normal human mammary epithelial cells [83]. 

DNMTs can contribute to tumorigenesis through CpG island-hypermethylation-mediated gene inactivation. DNMT1, DNMT3A and DNMT3B were substantially over-expressed in human hepatocarcinogenesis accompanied by a marked increase of tumor suppressor genes methylation [84]. DNMT1 is thought to be responsible for most of the abnormal promoter methylation in cancer cells. In particular, Robert *et al*. [85] demonstrated that DNMT1 was necessary and sufficient to maintain CpG island methylation and aberrant gene silencing in human cancer cells. It has been demonstrated that DNMT1 accounts for the majority of *de novo* methyltransferase activity in protein extracts from human colon cancer cells [86]. Taking into account the low intrinsic *de novo* methyltransferase ability of DNMT1 cases [6, 77], this protein may also be capable of initiating aberrant CpG island methylation patterns, replacing the conventional *de novo* DNMT3s, at least in cancer cells. 

Human cancer cells may differ in their threshold requirement for DNMT1. In particular, DNMT1-deficient breast cancer cells were not able to maintain promoter hypermethylation and exhibited decreased cell viability, whereas colorectal and bladder cancer cells were not affected [87]. This differential dependence on DNMT1 in maintaining cancer cell gene promoter hypermethylation and gene silencing may involve cooperativity between DNMTs. Genetic disruption of both DNMT1 and DNMT3B resulted in greater than 95% reduction in genomic cytosine-5 DNA methylation and re-activation of silenced tumor suppressor genes [88].

### 3.2. DNMTs, Chromatin Remodelling, Gene Silencing

The amino terminus of DNMTs was shown to function as a transcriptional repressor through interaction with a range of chromatin-associated proteins. That DNMTs and chromatin modification are tightly associated is suggested by a study where the H3K9 methyltransferase ESET, which contains a methyl-binding domain, was shown to interact with the oncogenic transcription factor ERG [89]. In addition, ESET was found to associate with HDAC1/2 and interact with the transcription co-repressors mSin3A/B [90]. ESET could participate in transcriptional regulation by methylating both DNA and histones and by recruiting other chromatin remodeling activities.

**Fig. (3):**
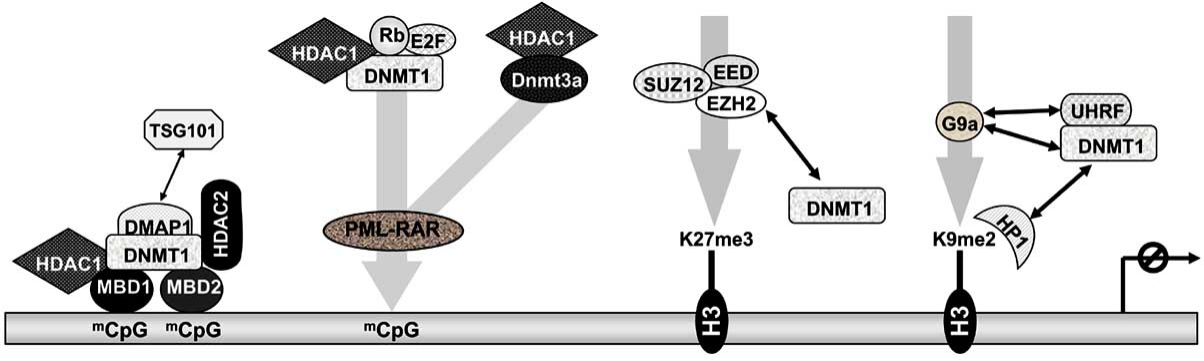
Model for the participation of DNMTs in chromatin modification and gene silencing (see text for details).

How are DNMTs targeted to specific regions of the genome? In leukemic cells, Dnmt1 and Dnmt3a are recruited by the oncogenic transcription factor PML-RAR to hypermethylate gene promoters. This is the first demonstration of recruitment of DNMTs by a transcription factor [91] (Fig. **3**). Moreover, DNMT1 is physically associated with the retinoblastoma (Rb) tumor suppressor gene product, and is targeted to a specific gene set through its interaction with the sequence-specific DNA-binding factor E2F [92]. However, transcriptional repression by DNMT1 at an E2F-responsive promoter is independent of its methyltransferase activity [92]. Furthermore, transcriptional co-repression by Dnmt3a at RP58-responsive promoter does not require its *de novo* methyltransferase activity. Like other co-repressors, Dnmt3a associates with HDAC1 through its ATRX-homology domain [93] (Fig. **3**). In addition, in colocteral carcinoma, histone modification and silencing of *p16^INK4a^* occurs prior to DNA methylation suggesting that DNA methylation serves as a “lock in” mechanism rather than initiator of gene silencing [94]. 

How are the transcriptionally inactive chromatin states maintained following DNA replication? DNMT1 was shown to form a complex with DMAP1 (DNMT1 associated protein) and HDAC2 [95] (Fig. **3**). DMAP1 which is sequestered to replication foci throughout S phase by DNMT1, was shown to directly interact with TSG101 (tumor susceptibility gene 101), a transcriptional co-repressor. In contrast, HDAC2 was demonstrated to bind to DNMT1 only during late S phase. The MBD2-MBD3 complex which interacts with DNMT1 in the late S phase can serve as a genome-wide scanning apparatus which searches hemimethylated DNA concurrent with DNA replication. Both, DNMT1 and MBD2-MBD3 have been shown to interact physically with HDAC1 [96] (Fig. **3**). 

Histone methyltransferase-mediated DNA methylation is another mechanism of gene silencing in cancer cells. The histone methyltransferase G9a, which catalyzes the dimethylation of H3K9, is considered to be responsible for the maintainance of transcriptionally silenced tumor suppressor gene promoters [97, 98]. During DNA replication, G9a through direct interaction with DNMT1 [99] and the DNMT1 co-factor UHRF1 [100] coordinates the transcriptional regulation of cancer-associated genes. Besides, the methylated H3K9- binding protein HP1 interacts with DNMT1 to mediate transcriptional repression of the anti-apoptotic *survivin* gene [101]. Of considerable interest, there is a mechanistic link between Polycomb-mediated repression and CpG methylation. The Polycomb Repressive Complex 2 (PRC2), which consists of EZH2, EED and SUZ12, is necessary along with DNMT1 to maintain epigenetic silencing of the pro-apoptotic *Fas* gene in cancer cells [102]. EZH2 which catalyzes the trimethylation of H3K27 is highly expressed in metastatic prostatic cancer and in lymphomas [103]. EZH2 was found to interact directly with DNMT1 for the coordinated and heritable transmission of CpG methylation patterns through DNA replication [104] (Fig. **3**). 

Most of the genome contains tight, transcriptionally repressive chromatin characterized by heavy CpG methylation and hypoacetylated histones that is replicated in late S phase. Thus, DNMTs and chromatin modifying enzymes could function in a cooperative manner for the faithful propagation of transcriptionally repressed chromatin states and stable gene silencing following cell division and DNA replication in cancer. In this way, it is ensured that the newly assembled nucleosomes are made up of densely methylated CpG dinucleotides and permissive histone marks; alternatively, deacetylase activity may be necessary to first remodel chromatin to allow DNMTs to carry out their methylating functions. 

Altogether, DNMTs can repress transcription independently of their ability to catalyze the methyltransferase reaction. DNMTs, apart from mediating DNA methylation, can serve as scaffolds to direct other chromatin modifying activities to establish a transcriptionally repressed chromatin structure.

### 3.3. DNMTs as Targets for Epigenetic Therapy in Cancer

The potential to reverse epigenetic modifications and up-regulate genes important to prevent or reverse tumorigenesis has become a new therapeutic target in cancer treatment [105]. In particular, DNMT inhibitors have been shown to reactivate expression of tumor suppressor genes that have undergone transcriptional epigenetic silencing [106] However, a major drawback of DNMT inhibitors is that they disrupt essential methylation at certain regions and they cause global hypermethylation which, as was discussed in the preceding section, is associated with great genomic instability. Another disadvantage is the toxicity to normal cells which is usually observed when higher doses are used [43]. 

The most extensively studied DNMT inhibitors are 5-azacytidine (Vidaza) and its deoxy analog, 5-aza-2’-deoxycytidine (Decitabine) which inhibit DNMTs and cause global hypomethylation [107]. Both are nucleoside analogues replacing cytidine and 2’-deoxycytidine, respectively, but they contain a nitrogen in the place of pyrimidine carbon at the position-5. These nucleoside analogues are incorporated only into replicating DNA, and they are therefore active only during S-phase. They serve as irreversible inhibitors of DNMTs, once incorporated into DNA, since they form covalent complexes with DNMTs leading to the depletion of active enzymes. This results in the demethylation of DNA in the progeny cells. 5-azacytidine is primarily activated by uridine-cytidine kinase and is partly incorporated into RNA, thus interfering with protein translation; 5-aza-2’-deoxycytidine is activated by deoxycytidine kinase and is only incorporated into DNA, thereby causing more efficient inhibition of DNMTs. Both drugs have been successfully used in clinical trials, especially in the field of haematological malignancies, such as myelodysplastic syndromes (MDS) and acute myeloid leukaemia [108, 109]. These azanucleosides restored epigenetically silenced tumor suppressor genes, like *p15^INK14^*, by demethylating their hypermethylated promoters [110]. Nevertheless, these DNMT inhibitors have some disadvantages. Both agents are quite toxic both *in vivo* and *in vitro*, and they are unstable in aqueus solution, which makes it difficult to administer both experimentally and clinically [111]. 

A chemically stable cytidine analog, zebularine [1-(beta-D-ribofuranosyl)-1,2-dihydropyrimidin-2-one], which exhibits a minimal cytotoxicity both *in vivo* and *in vitro* [112] and a high selectivity towards cancer cells was developed [113]. Zebularine contains a 2-(1H)-pyrimidone ring that was initially developed as a cytidine deaminase inhibitor, because it lacks the amino group on position carbon-4 of the pyrimidine ring [114]. Similar to the two azanucleosides, zebularine has been demonstrated to form a tight, covalent bond with bacterial methyltransferases at their active site [115]. The agent caused complete depletion of DNMT1 and partial depletion of DNMT3A and DNMT3B [113]. 

Several additional inhibitors of DNMTs have been described. The local anaesthetic procaine and its derivative procainamide, which is used to treat cardiac arrythmias, inhibit DNMTs by disturbing interactions between the proteins and their target sites [111]. The antihypertensive compound hydralazine has been shown to have demethylating activity which is probably attributed to the interaction between its nitrogen atoms and the DNMT active site [116]. In addition, natural products derived from green tea, epigallocatechin-3-gallate (EGCG), and from sponges, psammaplins, have been shown to inhibit DNMT activity by binding to and blocking the active site of proteins [117]. 

RG108 is a small molecule that specifically fits into the active site of human DNMT1 and renders the enzyme inactive, inducing demethylation and reactivation of tumor suppressor genes, without affecting the methylation status of the centromeric satellite sequences. These features make RG108 a promising candidate DNMT inhibitor, designated to block the active site of DNMTs and avoiding at the same time the adverse effects caused by global hypomethylation [118]. Furthermore, compounds specific for a particular DNMT have been developed. For instance, MG98, is an antisense oligonucleotide which inhibits human DNMT1 [119]. MG98 can exert its effect by specifically hybridizing to DNMT1 mRNA which may prevent translation, RNA transport or splicing. The antisense effect can be also mediated by activation of RNAseH, a ubiquitously expressed endonuclease which hydrolyzes the RNA strand of the heteroduplex [120].

MicroRNAs (miRNAs)-non-coding RNAs of 19 to 25 nucleotides in length that regulate gene expression through sequence-specific base pairing on the 3’ untranslated regions of target mRNA with subsequent translational inhibition or mRNA degradation [121]-were shown to target DNMTs. In particular, in lung cancer, the direct targeting of *DNMT3A *and *DNMT3B* by the miR-29 family was found to promote aberrant DNA hypomethylation and reactivation of the hypermethylated tumor suppressor genes *FHIT* and *WWOX *[122]. It was also demonstrated that miR-29b induced reexpression of the silenced tumor suppressor genes *p15^INK4b^* and *ESR1* in acute myeloid leukemia through direct targeting of *DNMT3A* and *DNMT3B* and indirect targeting of *DNMT1* [123]. These results, therefore, suggest a potential development of miRNA-based approaches for efficiently targeting DNMTs in malignant cells. 

As was discussed previously, there is an interdependent relationship between DNA methylation and chromatin modifying proteins. This association between the epigenetic pathways encouraged the development of combinatorial therapies that target multiple components of the epigenetic machinery. It was demonstrated that DNA and HDAC inhibitors act synergistically to reactivate tumor suppressor genes [124-126]. The DNMT inhibitor 5-aza-2’-deoxycytidine was also observed to reduce G9A and H3K9 dimethylation levels leading to reactivation of metastatic suppressor genes [127]. Moreover, it was shown that HDAC inhibitors decreased stability of DNMT3B mRNA and down-regulation of *de novo* DNA methyltransferase activity in human endometrial cancer cells [128]. The HDAC inhibitor depsipeptide induced reactivation of a variety of tumor suppressor gene promoters in lung, colon and pancreatic cancer cells by inhibiting expression of DNMT1 and G9a [129]. 

## 4. CONCLUDING REMARKS

Cancer is a polyepigenetic disease. Aberrant epigenetic silencing of tumor suppressor genes by CpG island promoter hypermethylation plays an important role in the pathogenesis of cancer. This review has focused on DNA methylation aberrations and the proteins that catalyze the methylation process in tumor cells. The potential reversibility of CpG island hypermethylation and re-activation of tumor suppressor and pro-apoptotic gene expression along with key control pathways in cancer cells presents attractive clinical possibilities. DNMT inhibitors represent promising anti-tumor therapeutics. The tumor profiles of CpG island hypermethylation provide useful biomolecular markers which allow the design of anti-cancer drugs that specifically target certain hypermethylation-silenced genes. Additional studies are required for further unravelling the role of DNMTs in the regulation of cancer-relevant genes expression. 

## Figures and Tables

**Table 1:** Genes Silenced by Promoter Hypermethylation

Pathway	Genes
Cell cycle regulation and apoptosis	p53, p73, Rb, p16^INK4a^, p15^INK4b^, p14^ARF^, HIC1, DAPK1, Caspase-8, APAF1, TMS-1 and RASSF1A
DNA-damage repair	O^6^-MGMT, hMLH1, GSTP1 and BRCA1
Tumor-cell invasion and metastasis	TIMP-3, APC, E-cadherin, H-cadherin, LK-B1, TSP-1, THBS-1, SFRP1, and VHL
Growth-factor response	SOCS-1, RARβ, AR, ER, PR, and PRLR

*APAF1*, apoptosis-associated factor 1; *APC*, adenomatous polyposis coli; *AR*, androgen receptor; *BRCA1*, breast carcinoma 1; *DAPK1*, death-associated protein kinase 1; *ER*, estrogene receptor; *GSTP1*, glutathione-S-transferase P1; *HIC1*, hypermethylated in cancer 1; *O^6^-MGMT*, *O^6^*-methylguanine-DNA methyltransferase; *PR*, progesterone receptor; *PRLR*, prolactin receptor; *RARβ*, retinoic acid receptor beta; *RASSF1A*, RAS-associated-domain family protein 1A; *Rb*, retinoblastoma; *SFRP1*, secreted frizzled-related protein 1; *SOCS-1*, suppressor of cytosine signaling-1; *THBS-1*, thrombospondin-1; *TIMP-3*, tissue inhibitor of metallopreoteinase 3; *VHL*, von Hippel-Lindau.
